# What Is Precision Psychotherapy?

**DOI:** 10.1093/jmp/jhag007

**Published:** 2026-03-18

**Authors:** Adrian Kind, Sascha Benjamin Fink, Henrik Walter

**Affiliations:** Research Training Group 2386 “Extrospection”, Humboldt Universität zu Berlin, Berlin, Germany; Center of Philosophy and AI Research, Friedrich-Alexander-Universität Erlangen-Nürnberg, Erlangen, Germany; Klinik für Psychiatrie und Psychotherapie, Campus Mitte, Charité – Universitätsmedizin Berlin, corporate member of Freie Universität Berlin and Humboldt-Universität zu Berlin, Deutsches Zentrum für psychische Gesundheit (DZPG), Berlin, Germany

**Keywords:** precision, precision medicine, precision psychiatry, precision psychotherapy, psychotherapy

## Abstract

Precision medicine impacts virtually all medical specializations, including psychiatry. Although precision psychiatry, in general, is a flourishing area of research and debate, psychotherapy as one pillar of psychiatry receives little attention. Theoretical discussions about precision psychotherapy are rare; research on precision in psychotherapy is just evolving. In this paper, we provide a conceptualization of precision psychotherapy providing a common idea of what should be understood as precision in the context of psychotherapy and what kind of psychotherapy research is considered to contribute to the aims of precision psychotherapy.

## I. INTRODUCTION

Ten years after the decade of the brain, Thomas R. Insel, then head of the National Institute of Mental Health, reflected on the enduring impact those years had on the field of psychiatry. According to Insel, the primary progress during the “decade of the brain” did not lie in specific research achievements but, rather, in the methodological advancements in biomedical research, the full potential of which would manifest in the subsequent years.^1^ Indeed, the emergence of precision psychiatry, capitalizing on these methodological advances, seems to validate his foresight (Walter, 2013). In contrast, other medical domains, particularly pharmacogenomics for rare diseases and cancer, were quicker to harness technological advancements to enhance the prediction, prevention, diagnostics, and treatment of illnesses in an individualized manner (Collins and McKusick, 2001; Spear et al., 2001; Hamburger and Collins, 2010; Jones, 2013; Carrasco-Ramiro, Peiró-Pastor, and Aguado, 2017; Lemoine, 2017).^2^ However, precision psychiatry gained momentum a few years later with the initiation of the RDoC Project (Insel, 2014). It is within this context that we encounter the initial mentions of the idea of ‘precision medicine for psychiatry’ and “precision psychiatry” (Vieta, 2015), accompanied by its first extensive conceptual discussion (Fernandes et al., 2017). Subsequently, research on precision psychiatry has been featured in edited collections (e.g., Baune, 2019; Passos et al., 2019; Williams and Hack, 2021), across major psychiatric journals, and in specialized publications like *Personalized Medicine in Psychiatry* (first issue 2017) or *Personalized Psychiatry and Neurology* (first issue 2021).^3^^,^^4^

It is intriguing to note that not all domains within psychiatry have garnered equal attention in transitioning toward precision medicine. Although strides have been made in diagnostic enhancement through computational phenotyping (e.g., Patzelt et al., 2018) and promising research in genetically informed psychopharmacology (e.g., Butler, 2018), one fundamental aspect of psychiatric treatment remains relatively unexplored in theoretical discourse and has seen a limited number of empirical investigations: psychotherapy.

To address this gap, particularly in theoretical discourse, we propose a conceptualization of “Precision Psychotherapy.” This endeavor aims to enrich the systematic exploration of the diverse sub-fields within precision medicine. We recognize that the significance of this undertaking extends beyond mere systematic exploration; it holds value in facilitating informed decision-making. Providing a conceptualization of precision psychiatry offers valuable guidance to researchers and stakeholders in medical research and services, aiding in non-trivial decision processes. For instance, it can inform decisions regarding which research to include in reviews within the field of precision psychotherapy or determine the type of research and practices worthy of funding in projects aimed at advancing the implementation and research of precision psychotherapy. Such decision-making necessitates criteria for distinguishing precision psychotherapy and its associated research.

Our approach to formulating a viable definition of precision psychotherapy involves beginning with a broad comprehension of precision medicine and tailoring this understanding to accommodate the specific context of psychotherapy. This process will yield a nuanced understanding of precision psychotherapy, aligning it with the broader concept of precision medicine while capturing the unique attributes of psychotherapy. However, a significant challenge in this endeavor is the nebulous nature of precision medicine as a concept. As highlighted by several authors, “there is no officially recognized definition” for precision medicine (Jain, 2015, 1). In many contexts, it simply serves as “an expression for describing a methodologically heterogeneous field of different approaches that are only loosely connected by a very general objective” (Langanke et al., 2015, 26). Given this ongoing debate and lack of a clear definition for precision medicine, we recognize the need for foundational and clarificatory groundwork on precision medicine before narrowing down its application to the domain of psychotherapy. The structure of the paper unfolds as follows.

In Section II, we initiate our discussion by delving into the sources and types of informational means typically utilized in precision medicine, extending this analysis to encompass precision psychotherapy. This examination stems from the endeavors of philosophers who have sought to define precision medicine by considering the nature of the information that informs it. Within contemporary philosophical dialogues concerning precision medicine, we observe the utilization of a broad and narrow conceptualization of precision medicine. The former asserts that precision medicine is solely grounded in microbiological and physiological data, while the latter extends its scope to encompass psychological, social, and environmental data as well.^5^ Although we refrain from committing to either stance for medicine at large, we advocate for adopting a broad understanding of the informational sources essential for achieving precision, particularly in the case of psychotherapy.

Doing so, we move on to Section III, where we contend that an adequate understanding of precision medicine or psychotherapy cannot be derived solely from identifying the relevant types of information used. Consequently, we pivot to explore how the aim of this research—an enhancement of precision through these information sources—can be comprehensively understood beyond the slogan to provide “the right patient with the right treatment at the right time” (e.g., Haldorsen, 2003; European Parliament Research Service, 2015; US Food and Drug Administration Service, 2018; Blackstone, 2019). A slogan does not meet the requirements of conceptual scrutiny we expect from a serious definitional attempt, because it only states the *purpose* of precision, does not explain not what precision is or how to achieve it. Furthermore, some areas of medicine may not only be concerned with the treatment or aim for an increase of precision in diagnostics. We argue for the idea that precision in medicine should be understood mainly as a reduction in indeterminacy more precisely semantic and epistemic indeterminacy.

Having presented our considerations regarding the appropriate understanding of precision in the context of medical research, we proceed to Section IV, where we substantiate the claim that precision entails the reduction of epistemic and semantic uncertainty in line with current medical research. We achieve this by examining the three key progress trajectories in medicine: Prediagnostic, Diagnostics, and Prognostics/Theranostics. We offer examples showcasing advancements in precision within each of these trajectories.

In Section V, integrating our considerations and applying them to the case of psychotherapy, we present our proposed definition of Precision Psychotherapy. We conclude that

An item of research increases the precision of psychotherapy if and only if it reduces a type of indeterminacy (i.e., reduces the range of plausible options) relevant for treating psychiatric illnesses (i.e., theranostics). It may do so by (a) refining psychiatric concepts and categories (reducing semantic indeterminacy), (b) providing evidences for or shifting certainty in line with psychiatrically relevant facts (reducing epistemic indeterminacy), or (c) increasing the likelihood with which a specific outcome is achieved by some intervention (reducing causal indeterminacy). For this, any type of information that reliably reduces such indeterminacies is suitable. Accordingly, precision psychotherapy as practice is realized where such reductions of indeterminacy are used in the psychotherapeutic treatment of patients.

Moreover, we provide an example showing how psychotherapy is more precise in this sense and may differ from current common psychotherapeutic practice (Section VI). Finally, in Section VII, we summarize by affirming that our defined conceptualization offers a valuable foundation for researchers and philosophers to engage in meaningful discourse about precision psychotherapy, transcending existing commonplace yet vacuous slogans.

## II. INFORMATIONAL SOURCES FOR PRECISION

The primary sources of information pivotal for the early progress in precision medicine originated from microbiological data. These insights initiated significant advancements in precision within oncology and the study of rare diseases (Collins and McKusick, 2001; Spear et al., 2001; Hamburger and Collins, 2010; Jones, 2013; Carrasco-Ramiro, Peiró-Pastor, and Aguado, 2017; Lemoine, 2017).^6^ As philosophers and scientists reflecting on the history of precision medicine have noted, “there have been a great many examples of interventions tailored to individual patient profiles, virtually all of them based on genetic profiles” especially “with genetically-mediated pharmacokinetic aspects of drugs” (Goetz and Schork, 2018, 5). Others, who concerned themselves with systematic reviews of the literature in the field, such as Schleidgen et al. (2013) did for “personalized medicine,” found that in research from the field we usually see attempts “to improve tailoring and timing of preventive and therapeutic measures by utilizing biological information and biomarkers on the level of molecular disease pathways, genetics, proteomics as well as metabolomics” (Schleidgen et al., 2013, 9).^7^

Due to this widely shared observation, some authors support a *narrow* biomedical conception of precision medicine, excluding various non-biological kinds of information. Schleidgen et al., for instance, claim that precision medicine

is not medicine with a special focus on the interests and preferences of the individual patient. For instance, PM does not include any reference to an adequate doctor–patient relationship. Hence, PM as such is not related to the term patient-centered medicine. (2013, 11)

A similar view is taken by Wiesinger, pointing out that

the personal characteristics of human beings—their self‐consciousness, their ability to reason and their rationality—are not even mentioned by personalized medicine in its search for molecular biomarkers. Personalized medicine refrains from the use of personal information, such as “psycho‐ and sociomarkers.” (2017, 2)

However, this is not the only position taken in the debate.

The National Research Council of the United States, for example, considers that precision medicine is exploiting the “variability in genes, environment, and lifestyle” (NRC, 2011, 1)—a view also supported by authors like Abttan and Welie, who emphasize that

more recently, personalized medicine has taken an important turn by expanding the number and type of health-related data used […] personalized medicine has moved beyond merely genetic data to include the full range of available patient information, from a molecular scale (proteome, transcriptome, metabolome, etc.) to an epidemiological one (foodome, sociome, environtome, etc.). (2020, 3)

In this view, the set of evidence used may also contain information about certain lifestyle factors such as the diet of subjects, their social situation (e.g., employments or friendships), and various environmental factors. Nothing in principle speaks against including “psychomarkers,” for example, information about certain psychological traits, such as personality traits. Taking into account all these factors, going beyond microbiology is what one might call the broad view, in contrast to the above-proclaimed narrow view focusing solely on biological information.

Both approaches, broad and narrow, are present in medical science and practice. Instances of a narrow perspective can be seen in the assessment of heritable genetic variations of subjects to determine whether they have a high risk of developing cancer (e.g., Shin, Bode, and Dong, 2017). On the other hand, recent developments in precision medicine focusing on type-II diabetes highlight the central relevance of lifestyle factors (e.g., Mutie, Giordano, and Franks, 2017). When considering the broad and narrow conceptualizations as competing positions, one may inquire about their suitability for psychiatry, particularly psychotherapy. Let us begin by examining psychiatry to determine whether, even at this level, a clear decision can be made in favor of one or the other source of information being more applicable.

Looking at existing discussions in precision psychiatry, we can observe both narrow and broad focuses. The narrow perspective is embraced by the author who coined the term “precision psychiatry.” He anticipates that, as precision psychiatry advances, “nobody will be able to work denying the biological substrate of mental diseases” (Vieta, 2015, 117). This narrow emphasis on biological factors is shared by proponents who envision precision psychiatry concentrating on “Multi-omics, neuroimaging, big data and high-density data approaches, exposome and molecular epidemiology and physiology [that] should converge towards specific biomarkers” (Köhne and Van Os, 2021, 1409).

However, other authors explicitly adopt a broader understanding, such as Fernandes et al. (2017), who acknowledge that precision psychiatry “rests on, and simultaneously contributes to, the evolving knowledge of the biological pathways involved in the major mental illnesses,” ultimately leading to an “appropriately integrative understanding of mental illness as disorders of the brain” (Fernandes et al., 2017, 2). They make it clear that this appropriately integrative approach entails incorporating environmental and lifestyle factors into the modeling process. Similarly, Baune highlights that precision psychiatry is an approach “that takes into account individual variability of genes, environment, and lifestyle” (2019, 1). Furthermore, he emphasizes that in the process of researching and applying precision medicine, consideration is given to “individual clinical characteristics of a patient,” encompassing “neurocognitive, affective, and functional profiles,” as well as the “personality, insight, and resilience” of patients (Baune, 2019, 2).

Shifting attention from medicine in general to psychiatry does not definitively resolve the debate between the narrow and broad understanding, as both continue to be applied. Consequently, the resolution of this issue needs to occur at the level of psychotherapy itself.

When would it, at least prima facie, be more plausible to assume the narrow or broad notion for the context of precision psychotherapy? The narrow notion would be *prima facie* plausible if efforts related to increasing the precision of psychotherapy should mainly rely on the use of biological information about patients. On the other hand, the broad notion would be prima facie more plausible if, in addition to the possibility that biological information might contribute to precision regarding psychotherapy, other information—such as neurocognitive, affective and functional profiles, personality traits, social information, and clinical characteristics of these patients—may also contribute to an increase in precision.

To settle whether precision medicine in general should be understood more plausibly along the lines of the wide or the narrow notion is a complicated matter. Concerns about the inherent tension of including psychomarker data in precision medicine (including non-psychomedical fields such as cardiology) may arise. Moreover, including environmental and epidemiological data may raise questions about how precise these recommendations can be based on the vastness of this information. Because these concerns lie beyond the scope of this paper, we remain neutral on this issue with respect to medicine in general. Instead, we just focus on our main concern, psychotherapy. A brief look at psychotherapy research shows us that, at least up until now, we find examples supporting the apparent possibility to increase precision based on both biological and non-biological information. Let us illustrate this.

As an example of the use of biological information, we can consider the burgeoning understanding of the impact of psychotherapy on brain structures and functioning (see Beauregard, 2014). Although this field of psychotherapy research is still relatively young, several promising examples have emerged. For instance, Colvonen et al. (2017) highlight that research suggests pre-treatment biomarkers (specific functional neural systems, glucocorticoid sensitivity and metabolism, heart rate, gene methylation, and certain genotypes associated with serotonin and glucocorticoids) can predict a positive response to psychotherapeutic treatment of PTSD. Riess (2011) points out that the quality of the therapeutic relationship between therapist and patient, assumed to be a relevant mediator or moderator of positive outcomes in psychotherapy, can be linked to (dis)concordances in the autonomic nervous system arousal in therapists and patients during therapy sessions. This is manifested by heart rate, respiration rate, muscle tension, galvanic skin resistance, electroencephalography, and brain imaging markers. Additionally, Marceau et al. (2018) found in their review that biomarkers (specifically hypoactivation in prefrontal and cingulate regions) can predict treatment response to psychotherapeutic interventions for patients suffering from Borderline Personality Disorder. These examples showcase the relevance of biological information in increasing the precision of psychotherapeutic treatments. However, we also find instances where non-biological information plays a crucial role.

Examples of cases where the use of non-biological information increases the precision of psychotherapy are numerous, constituting the standard information that has been crucial in psychotherapy research over the last decades. Here are some illustrations: The NIMH Treatment of Depression Collaborative Research Program found that baseline severity of depression predicts differential treatment effects. Generally, psychopharmacological treatments appear to be more effective than psychotherapy in severely depressed patients, while there is no difference found between pharmacotherapy and psychotherapies in subjects who are less severely depressed (Elkin et al., 1995). Another example pertains to research on personality traits and their role in the recovery from depression. It was revealed that the Big Five personality trait of neuroticism does not moderate the effects of CBT in treatment-resistant individuals (Spek et al., 2008; Fournier et al., 2009; Wiles le al., 2014). Nor did dysfunctional attitudes and metacognitive awareness (Wiles et al., 2014), outcomes that discourage the practice of patient selection for CBT based on these features. Further, research highlights the significance of patients’ preferences regarding the type of psychological intervention they receive on dropout rates in treatment and the magnitude of treatment effect. Aligning treatment with patient preferences significantly improves dropout rates, as well as clinical outcomes (Swift et al., 2019; Windle et al., 2019). Lastly, in a more recent example, work by Saunders, Buckman, and Pilling (2020) analyzed routine data from 44,905 patients who received treatment. They linked patterns of recovery under different treatments (low intensity treatments—e.g., facilitator-led/guided self-help or computerized-CBT, and high-intensity treatments—e.g., CBT, interpersonal therapy (IPT), or counseling) and discovered clusters of patients responding differentially to treatments in terms of reliable recovery, improvement, and clinical deterioration. The information consistently used encompasses clinical measures (depression severity, anxiety severity, functional impairment, phobia self-rating), medical information (medical prescriptions), biological information (age at referral, sex), and social information (ethnic group, welfare status).

Considering all the above examples of the use of both broad and narrow information to support claims about how to more specifically select and target therapeutic interventions, it leads to a potential resolution of the debate regarding which types of information should be considered as sources of information for precision psychotherapy. If, as it prima facie appears, all the examples of psychotherapy research mentioned in the preceding paragraphs would qualify as research increasing the precision of psychotherapy, it would be appropriate to adopt the broad notion of information rather than the narrow one. This is because the narrow notion would only cover part of the described sources of information, while the broad one would encompass all the sources of information discussed above. However, whether these examples indeed qualify as increasing precision can only be answered once we establish our understanding of precision, a task we undertake in the next section. After presenting our understanding of precision, we briefly revisit the above examples and demonstrate retrospectively that the antecedent of our presented conditional is indeed true.^8^

## III. PRECISION FOR MEDICINE

When discussing precision medicine, and precision psychotherapy in particular, the question of how ‘precision’ in this context should be understood is natural and pivotal. There are several ways to conceptualize precision, broadly falling into two larger groups: the technical sense of precision within metrology, the science of measurement, and several commonsensical understandings that can be elaborated based on existing philosophical work on indeterminacy. However, we contend that the metrological account is inadequate in this context, and we should instead opt for a philosophically informed account. To establish the plausibility of this assertion, let us first delve into the metrological option and its inherent problems.

The International Vocabulary of Metrology (2021) defines precision as the proximity of several measurements on the same target. However, these measurements must only be close to each other, not necessarily to the true value. In this sense, one can be precise but wrong, highlighting the distinction between precision and accuracy, which refers to the closeness between the measured value and the true value. For instance, in a game of darts, you are accurate if your darts hit the bull’s eye, and the degree of accuracy decreases as your darts land farther away from the bull’s eye. Nevertheless, they might still cluster tightly if you throw precisely but inaccurately. It is precisely this independence of precision from accuracy, allowing for an increase in precision without a corresponding increase (and potentially even allowing a decrease) in accuracy, that renders the metrological understanding of precision unsuitable for precision medicine.

If the “very general aim” (Langanke et al., 2015, 26) of precision medicine assumed by stakeholders is encapsulated in the slogan “the right treatment for the right patient,” it broadly encompasses what precision medicine is partly about. In this context, the metrological understanding of precision, as defined by the International Vocabulary of Metrology, seems unsuitable for precision medicine. If precision in medicine (and in psychotherapy as well) is indeed about determining the most appropriate (rather than inappropriate) treatment, it necessitates accurate judgments about potential treatment outcomes based on the specificities of patients. Mere metrological precision, where the measurements are close to each other but not necessarily to the true value, is inadequate. Simply arriving at the same conclusion regarding the treatment (precision) does not guarantee the correct treatment (accuracy). Epistemic progress in precision medicine should be intrinsically linked to an increase in accuracy. This progress should lead to a better understanding of how factors such as the specificities of a patient’s condition influence different treatment outcomes. Therefore, whatever definition of precision in medicine is ultimately adopted, it should at the very least provide reliable, more accurate insights into medical facts that influence treatment outcomes. Epistemic progress, aiming for more accurate insights into medical matters, would not be guaranteed if a meteorology-based notion of precision were adopted to define precision medicine or psychotherapy.

Beyond the narrow confines of metrology, achieving precision is often associated with a reduction in some form of indeterminacy. This principle also extends to the “precision” in metrology: the more precisely I throw darts, the more definite it becomes where they will land on the board after each throw. At the pinnacle of precision, all indeterminacy vanishes, and each throw hits the same point. But what exactly is indeterminacy? In a general sense, something, x, is considered indeterminate (to someone, to everyone, or in itself), if it is not obvious which of several options concerning x is correct. When our throws lack precision, it remains unclear where they will land. As we strive for precision, we can progressively eliminate potential landing sites on the board. By broadening our perspective beyond metrology, we may arrive at an understanding of precision in terms of reducing indeterminacy that aligns more suitably with precision as pursued in medicine. This leads us to the alternative version, namely, the more commonsensical, philosophically informed understanding of precision that we propose to adopt.^9^

Commencing with the common-sense understanding of precision as the reduction of indeterminacy, we can delve deeper into comprehending indeterminacy and its reduction by categorizing the various types of indeterminacy and identifying those relevant in the context of medicine. The types of indeterminacy we differentiate are *absolute* and *relative* indeterminacy. Absolute indeterminacy is further subdivided into *ontological* and *causal* indeterminacy, while relative indeterminacy is categorized into *semantic* and *epistemic* indeterminacy. Subsequently, we argue that what primarily concerns medicine is semantic and epistemic indeterminacy and its subsequent reduction.

Something can be indeterminate with respect to something, for example a specific group, method, or framework, which we can denote as relative indeterminacy. Conversely, something can be objectively or inherently indeterminate, which we categorize as absolute indeterminacy. For example, the next number on a roulette wheel may be indeterminate to an individual, but given the metaphysical macroconditions of the ball and the wheel, it is objectively determinable. Physics narrows down the possibilities to a single outcome, but from the individual’s perspective, all options remain uncertain. In this case, it is indeterminate relative to the individual but not in itself. This scenario exemplifies relative indeterminacy. In contrast, consider Schrödinger’s cat in quantum mechanics: before observation, it is neither definitively dead nor alive. According to the Copenhagen interpretation of quantum physics, the status of Schrödinger’s cat is inherently indeterminate. This is an instance of absolute indeterminacy, also referred to as metaphysical indeterminacy.

*Prima facie*, it seems that metaphysical indeterminacy and attempts to reduce it for the sake of precision might not be readily applicable in the field of medicine. Considering the ontological aspect, which pertains to the indeterminacy of whether something exists or not, it does not seem that medicine has a significant role here. The objective of medical diagnosis is to ascertain the existing values within a patient’s body, not to create these values. Physicians aim to determine the specific illness a patient has, rather than conjuring an illness into existence for a patient. Similarly, medical interventions aim to modify certain values within a patient’s body, not generate them from scratch. Consequently, a reduction of metaphysical indeterminacy in this ontological sense appears to be ill-suited for understanding precision medicine.

However, there is a metaphysical notion of indeterminacy that might be relevant, particularly considering that medical interventions involve a form of manipulation aimed at making things happen. In this context, we could consider a form of causal indeterminacy. Unlike the momentary metaphysical indeterminacy, which is concerned with a specific moment in time (e.g., the moment before we open Schrödinger’s box), causal indeterminacy pertains to the uncertainty regarding the outcome of a process. Stephen Jay Gould, for instance, suggested in *Wonderful Life* (1989) that evolution is causally indeterminate: if we were to rewind life’s tape, we might not witness the emergence of the same individuals or even species. Similarly, could there not be medical interventions in which the outcome is uncertain? And could an increase in precision not imply that we gain a better understanding of the potential outcomes of our intervention?

These questions pose distinct inquiries. The first delves into the realm of absolute causal indeterminacy, where the outcome of an intervention is de facto uncertain, beyond control, and unpredictable as an inherent aspect of the intervention process. However, interventions characterized by such inherent uncertainty in outcomes would be less preferable than those where outcomes can be controlled. In the realm of medicine, the goal is to minimize uncertainty and benefit the patient, making interventions with inherently unpredictable outcomes less desirable. If such interventions exist, they would be phased out in favor of less indeterminate ones, a trajectory dictated not only by precision medicine but by the fundamental objectives of medical practice.

On the other hand, the second question pertains to relative causal indeterminacy. Here, the indeterminacy in outcomes arises not from the process of intervention itself but from previously uncontrolled factors that intervene in the process, factors that can be identified and managed. Some of these influencing factors may have been previously unknown, hence not regulated. By identifying and bringing these factors into focus, they can be controlled to a certain extent. Consider the historical example of Ignaz Semmelweis, who increased control over maternal deaths during childbirth by recognizing and addressing the spread of materials from dissecting doctors to women in labor. The precision achieved in such cases stems from knowledge of and mastery over factors that intervene in the outcome of a process, rather than from the inherent indeterminacy of the process itself. In the context of medicine, precision primarily focuses on relative indeterminacy—relative to our current state of knowledge. It involves gaining knowledge and control over factors that intervene in outcomes, moving from absolute, metaphysical indeterminacy to the realm of relative indeterminacy.^10^

For relative indeterminacy, which, to recall, occurs when something is not inherently indeterminate but is, for example, specific to a particular group, method, or framework, we may differentiate between *semantic* and *epistemic* indeterminacy.

Considering semantic indeterminacy first, it arises when the meaning of a term or utterance is not fixed—it could signify this or that. Ambiguity, a prominent form of semantic indeterminacy, occurs when several meanings are concealed under homophones, as seen with “bank,” which may refer to a financial institution or the edge of a river. However, the semantic indeterminacy underlying ambiguous phrases can be resolved by clarifying intended meaning. By exposing and resolving ambiguity, we achieve greater precision. Nevertheless, addressing ambiguity is a straightforward objective for precision medicine. However, not all types of semantic indeterminacy can be nullified by exposing them.

Unlike ambiguity, indeterminacy associated with vagueness cannot be resolved by being exposed. A term is considered vague if it possesses both clear-cut and borderline cases. For instance, Yul Brunner is clearly bald, and Dave Grohl is not. However, when considering individuals like Woody Harrelson or Jason Statham, there is disagreement among competent speakers. For these borderline cases, it remains unclear whether the concept of baldness applies or not. Consequently, statements attributing the concept to such borderline cases become uncertain in their truth value. Therefore, vagueness is often interpreted as a form of semantic indeterminacy, where it is indeterminate what falls under a concept and whether a statement with a vague concept is true or not for certain borderline cases.

Another form of indeterminacy arises with increased generality and quantification. With either general or existential quantification (using terms like “all” and “”some”), we shift from referring to specific objects to speaking about everything within our universe of discourse. However, as our statements become more general, they also become less determinate. For instance, if I know that Bob is a builder, I have a clear idea of whom to contact to fix my wall. On the other hand, if I only know that *someone* is a builder, it may take me considerable time to identify the right person.

A third form of indeterminacy, associated with increased generality, arises from the subsumability of concepts, often discussed in terms of determinables and determinates. A concept is a determinable if and only if it subsumes several more specific concepts (its determinates). What makes a determinate-ascription true also makes the determinable-ascription true, but the truth of a determinable-description leaves open which determinate-ascription is true. For instance, if I know that your carpet is green, I know that it has a color, but I do not know whether it is forest green, moss green, teal, etc. So “green” is more determinate than “colored,” but less determinate than “teal.” Green is a determinate relative to the determinable “colored” but a determinable relative to more precise color concepts.^11^

If we consider these three forms of semantic indeterminacy, it appears that for the context of medicine, the reduction or elimination of vagueness of terms, as well as ambiguity, presents an adequate aim because they allow us to be more precise.^12^ However, there is also a catch: the concept “vague” is itself vague because there are borderline cases of borderline cases. We might not know where the night begins or where the day ends, so we group all of the borderline cases under “dusk.” That might make our use of “day” more precise, but it leaves open the question: Where does “dusk” begin and end? If vagueness is vague, so is its counterpart, precision. We can probably not eradicate vagueness fully in precision medicine—when exactly is blood pressure high? How long *exactly* do I have to be sad to count as depressed?—but we can rein it in more and more, which is already progress. Lets us next move to the other relevant form of relative indeterminacy, epistemic indeterminacy.

Epistemic indeterminacy deals with not knowing what is the case, even though the concepts are clear. It is sometimes understood as degrees of uncertainty regarding the truth of a proposition. As the mathematician De Finetti famously suggested, this shows itself behaviorally in the way we bet: The more certain we are that *x* happens, the more we are willing to bet on *x*, and vice versa. This fits well with the different areas of medicine: In diagnostics, we may want to develop more fine-grained tools to know more about the status of a patient; and in therapy, we may want to develop more fine-grained interventions or learn more about intervening variables or underlying mechanisms in order to be more certain about producing specific outcomes.

Since medicine, as it is practiced today, is evidence-based and therefore guided by the empirical sciences, medical knowledge, like all scientific knowledge, must be considered fallible. We should expect precision medicine to aim at better methods to gather information and decrease uncertainty. However, achieving full precision or absolute certainty may not be a realistic goal. Instead, a reduction in uncertainty may suffice. According to the immutability account of certainty, advocated by Stanley (2008), one’s belief that proposition p is considered certain when it is justified to the highest degree. This idea traces back to Russell, who wrote that “a proposition is certain when it has the highest degree of credibility, either intrinsically or as a result of argument” (1948, 396). The challenge lies in defining what constitutes “the highest degree” of certainty.

As pointed out by Reed (2022), spelling out the phrase “the highest degree” is a complex task. If we interpret it to mean “justified to the highest degree” in comparison to all other beliefs an individual holds regarding a subject matter, the notion of the highest degree will vary, based on the other beliefs the subject holds, which might be relatively low. This could lead to the highest degree of certainty being low as well (i.e., I am certain of believing *p*, as I am certain of anything). The same issue could arise if we broaden the certainty comparison to the intersubjective field, considering the beliefs regarding a topic held by everyone else. It appears that we do not want a standard that is too relative. An elegant solution to this problem is offered by Chisholm:

*p* is certain for *S* = df for every *q*, believing *p* is more justified for *S* than withholding *q*, and believing *p* is at least as justified for *S* as is believing *q*. (1989, 12)

This definition of certainty ensures, on one hand, that a supported belief *p* is the belief that can be held with the highest certainty, surpassing all beliefs *q.* On the other hand, it also guarantees that p is supported to a significant degree. This is achieved by stipulating that supporting *p* must not only be more rational than withholding *p*, but also more justified than the evidence for propositions *q*, compelling us to withhold judgment about their truth. Such an understanding of epistemic precision in terms of certainty, as proposed by Chisholm, is suitable in the context of medicine. It avoids an approach to certainty that may *prima facie* appear incompatible with medical knowledge, such as understandings of certainty as being inviolable in the face of contrary evidence. Instead, it posits that certainty depends on degrees of evidence that sufficiently support a proposition, making it better supported than its alternatives to be certain of it. Consequently, there is room to discuss certainty resulting from varying degrees of evidential support. This approach aligns well with the practical handling of knowledge in medicine, which involves the accumulation of evidence of different sorts for or against certain medical propositions.

Summarizing our discussion on relative indeterminacy, semantic and epistemic indeterminacy, and their reduction in the context of medicine, the idea of increasing precision in medicine can manifest in two main forms. First, we can enhance the precision of medical terms through reducing vagueness, ambiguity, or uncertainty in their applications, aligning them more closely with medical objectives. Second, we can formulate medical propositions that can be held with a higher degree of certainty, or by elevating the certainty level we have for existing medical propositions.

It is important to note that these ways to increase precision in medicine are likely to interact regularly. Determining whether a novel formulation of a medical proposition or changes in a medical concept qualify as improvements (i.e., more precision) cannot be decided in the armchair; it often requires clinical expertise and empirical support for its adequacy. Conversely, some increases in epistemic precision might stem from using more fine-grained concepts. In conclusion, the strongest moments of precision increase, the ideal case if one will, would be one in which all aspects are present. Consider a historic example in Robert Koch, who discovered the etiological role of Mycobacterium tuberculosis as the cause of tuberculosis, establishing and supporting a new medical proposition regarding the cause and mode of transmission. Previous researchers like Villemin (1865) had already demonstrated that tuberculosis is an infectious disease by infecting rabbits with material from cadavers of individuals who died from tuberculosis (Villemin, 1865). However, Koch showed that it was a bacterium causing the outbreak and transmission (Kaufmann and Schaible, 2005). This increased semantic precision, as we transitioned from Villemin’s insight that something causes a tuberculosis infection to Koch’s understanding that Mycobacterium tuberculosis causes a tuberculosis infection. It increased epistemic precision as Koch provided evidence making this specific bacterium the most likely cause. Additionally, it increased causal precision of an intervention as a function of the increase of epistemic precision regarding insight into causes, allowing for a more targeted intervention to prevent tuberculosis infections.

To conclude this section, let us ask what exactly is the advantage of our push for the idea of precision in medical contexts being understood in terms of decreasing epistemic and semantic indeterminacy over the meteorological notion of precision. To put it simply, while the metrological idea of precision does bear no inherent relationship to truth-tracking (measures can become more precise without becoming more accurate), thinking of precision in terms of reducing semantic or epistemic uncertainty does. Decreasing semantic and epistemic indeterminacy seems to bring us closer to the right semantics, and adequate beliefs about medical matters. Furthermore, as we illustrate in the next section, precision improvements in medical research appear to be primarily achieved through reducing semantic and epistemic uncertainty, rather than through meteorological precision enhancements.

## IV. DIAGNOSTICS, PROGNOSTICS, AND THERANOSTICS

If we accept that an increase in precision in a medical field would mean a decrease in semantic or epistemic indeterminacy, one may complain that this is a rather abstract aim. But how shall this aim be realized? Let us considering the different domains of medical practice in which precision might be improved and look at some examples that may help. The areas we want to examine for this purpose will be called prediagnostic, diagnostics, prognostics, and theranostics.

The task of prediagnostic is to identify prodromal factors allowing prediction of the future occurrence of disease.^13^ Such factors might be its predictive markers or causal precursors. The latter case is especially interesting, because it provides avenues for early preventive interventions. Prediagnostic therefore falls into the domain of *Predictive Medicine* (Jen Shahrokhi, and Varacallo, 2015).

Increasing precision in this domain can occur by: (a) providing evidence for certain meaningfully predictive (and perhaps causally relevant) factors for a disease; (b) broadening the evidence base so that we can predict with greater certainty and less bias from a single source; (c) presenting evidence against a likely factor being predictive and minimizing its interference with our prediction; and (d) revising established hypotheses in light of incoming evidence to make the proposition more precise or probable.

Let us consider an example of a potential increase in precision, achieved by reducing generality. Take the hypothesis that higher overall leukocyte counts in women are associated with an increased risk of breast cancer. This proposition is unspecific regarding whether any specific leukocyte subtypes play a crucial role in breast cancer risk or not. Both identifying leukocyte subtypes and testing whether some are more relevant than others thereby specifies and precisifies the hypothesis. Doing so, this reduction of generality contributes not only to theoretical interest but also to practical interest—by homing in on more specific predictive factors.^14^ Let us proceed next to diagnostics.

The task of diagnostics is to identify current diseases in patients. Here, we may achieve greater precision by assessing the patient’s condition more accurately or by distinguishing between potential diseases, such as distinguishing tuberculosis from a bacterial infection. Precision can therefore be achieved by: (a) providing evidence for diagnostic markers that reliably identify a specific medical condition; (b) increasing the reliability of certain diagnostic markers; (c) providing evidence against certain factors being diagnostically relevant, minimizing their interference with determining medical truth; (d) revising diagnostic categories or factors based on empirical evidence, making them more precise or useful for diagnostics.

Let us consider, as an example, the lumping and splitting of diagnostic categories. Lumping of diagnostic categories (consolidating two or more into one) might not always increase precision, though it could still be beneficial. On the other hand, splitting often decreases ambiguity and vagueness. While for a long time, identifying disease entities in medicine relied on pure phenotype-based categorization, the rise of molecular biology has changed this practice in recent decades, prompting the medical community to consider both disease genotypes and phenotypes in determining disease entities (Biesecker, 1998). More recently, the Splitting and Lumping Working Group (SLWG) of ClinGen (2018) has made efforts to formulate the factors that should guide decisions to lump or split diagnostic categories and the procedures by which evidence should be weighed as reasons for lumping or splitting, including factors like phenotypical differences, molecular mechanisms of development, genetic basis, and inheritance patterns (Thaxton et al., 2022). An instance of kind splitting that would reduce ambiguity and therefore increase semantic precision in diagnostics discussed by the workgroup is the case of Multiple Endocrine Neoplasia 2 (MEN2). MEN2 is a type of cancer associated with mutations in the RET-Gene. This disease is considered to have two symptomatically distinct subtypes: MEN2-A (sometimes called Sipple-Syndrome) and MEN2-B (sometimes called Wagenmann-Froböse-Syndrome). Based on reviews of evidence on the genetic basis, phenotypical differences, molecular mechanisms of development, and inheritance patterns, the SLWG concludes that instead of considering the conditions named MEN2-A and MEN2-B subtypes of the same disease, they should be regarded as two different diseases altogether (ClinGen, 2018). This move, if accepted, increases the precision of diagnostic categorization by disambiguating MEN2, formally referring to two different disease entities as subtypes, into two entirely different conditions (ClinGen, 2018). Now let us turn to prognostics.

The task of prognostics involves identifying factors that enable predictions regarding how a patient might prevent, recover from, or potentially relapse into a certain disease, either with or without specific treatments.^15^ This is a facet of predictive medicine closely related to s*tratified medicine* (Bell, 2014), which aims to predict the interventions that yield the most benefits and have the fewest side effects for a reference group of patients based on prognostic factors. This ideally allows for more finely grained recommendations than those based solely on diagnostic categorization. The intervention may aim to prevent, cure, or at least improve a patient’s condition. When discussing the topic of treatment in this context, some authors prefer to frame it in terms of *theranostics*.

The domain of prognostics can be enhanced by: (a) providing evidence that prognostic factors, including the type of intervention, enable predictions about whether an intervention increases the chances of recovery, or prevents, offsets, or mitigates a disease’s outbreak, progression, or relapse; (b) increasing the reliability of predictive factors to serve the aims of (a); (c) providing evidence against some considered factors being predictive; (d) revising the conceptualization of predictive factors in light of empirical evidence. An example of increased precision in prognostics can be seen in research on the effectiveness of monotherapies for cancer (Adam et al., 2020). In this field, large-scale in vitro tests are conducted to associate molecular profiles of cancer cells with their response to drugs. The National Cancer Institute Developmental Therapeutics Program published data on the response of 60 cancer cell lines (known as NCI60) to tens of thousands of chemical compounds, including a large number of drugs. One important result of these efforts was the discovery of the 26S proteasome inhibitor bortezomib, now used in multiple myeloma treatment (Shoemaker, 2006). Since then, in vitro drug screens of cancer cell lines have become a popular approach to discovering the multiomic underpinnings of drug sensitivity and resistance.

While this section focused on the domains in which medicine might increase its precision in general, the next section focuses on bringing these ideas about semantic and epistemic uncertainty from the previous section to bear on the case of precision psychotherapy. Before we move on, there is a remaining argumentative depth from Section II that is still open. To recall: there, we claimed we would later show that the examples we used to plausibly support the wide, rather than the narrow, notion of psychotherapy to be used are indeed examples of research in psychotherapy that would qualify as increasing precision. This point is easy to make now, given our work in this and the last section.

The first examples, using biomarkers such as PTSD biomarkers for treatment outcome prediction (Colvonen et al., 2017), biomarkers for the prediction of the therapeutic relationship (Riess, 2011), and biomarkers for outcome prediction in borderline patients, increase epistemic precision in theranostics by establishing new medical propositions concerned with factors whose presence or absence predicts the success of therapeutic treatment outcomes. The same holds true for the findings from the NIMH Treatment of Depression Collaborative Research Program (Elkin et al., 1995). On the other hand, the mentioned research on relevant features of treatment-resistant depressive patients that showed several features to be irrelevant in predicting outcomes in therapy increased precision in theranostics by decreasing certainty in incorrect medical propositions (Spek et al., 2008; Fournier et al., 2009; Wiles et al., 2014). Similarly, the research on the relevance of patient preferences on dropout rates and outcomes in therapy contributes to increasing precision by providing evidence for a certain medical proposition falling into the trajectory of theranostics that would increase certainty in certain medical propositions (Swift et al., 2019; Windle et al., 2019). The same applies to the clustering studies for dividing patients into groups that benefit from low and high-intensity treatment. Perhaps they may find a helpful refinement in what we attempted to do by providing this definition, because it provides evidence that certain factors (e.g., gender, age, personality) of a patient predict some psychotherapeutic interventions’ potential to lead to recovery from a mental illness or, respectively, prevent, offset, or mitigate its outbreak, progression, or relapse (Saunders et al., 2020).

Now that it is clear that the examples used to support the broader notion of information in the context of psychotherapy indeed seem to increase precision, and thus the antecedent of our earlier claim in Section II holds true, let us take all our considerations from the last sections and apply them to the question of how we should define precision psychotherapy.

## V. DEFINING PRECISION PSYCHOTHERAPY

We have discussed the informational sources that should be considered as drivers of precision medicine or, respectively, psychotherapy in Section II. In Section III, we explored how to best conceptualize precision as an aim in precision medicine. We also provided a differentiation of general trajectories of progress in precision and showed examples of how progress, in line with our proposed understanding of precision, can be made in each of them in Section IV. In this section, we draw together our theoretical considerations from the last sections and apply them to arrive at a working definition of precision psychotherapy. Our proposal is as follows:

An item of research increases the precision of psychotherapy if and only if it reduces a type of indeterminacy (i.e., reduces the range of plausible options) relevant for treating psychiatric illnesses (i.e., theranostics).They may do so by (a) refining psychiatric concepts and categories (reducing semantic indeterminacy), (b) providing evidences for or shifting certainty in line with psychiatrically relevant facts (reducing epistemic indeterminacy), or (c) increasing the likelihood with which a specific outcome is achieved by some intervention (reducing causal indeterminacy). For this, any type of information that reliably reduces such indeterminacies is suitable. Accordingly, precision psychotherapy as practice is realized where such reductions of indeterminacy are used in the psychotherapeutic treatment of patients.

More precisely, this would mean that a research item contributes to precision psychotherapy if:

- It provides evidence that a certain factor (e.g., gender, age, personality) of a patient predicts the potential of some psychotherapeutic interventions to lead to recovery from a mental illness or, respectively, prevent, offset, or mitigate its outbreak, progression, or relapse.
- It increases the reliability and credibility of a predictive factor to be used in cases like (i).
- It provides evidence against some considered factor, thereby decreasing its potential to interfere with tracking medical truth.
- It revises the conceptualization of predictive factors in light of empirical evidence, making the medically relevant concepts more precise and testable, i.e., less ambiguous, vague, or general.

This definition, developed from our theoretical considerations on precision medicine in general and applied to the specific case of psychotherapy, offers two additional benefits beyond the reasoning we have presented so far to support it. The first benefit is that this understanding is in line with, but more general and detailed, than other suggestions in the literature. For example, the goal of precision psychotherapy is described as “increasing the success of psychological interventions by identifying predictors of response” (Martinez-Aran and Vieta, 2022). It also aligns with the mandate for individualization presented by Gordon Paul, which emphasizes finding the optimal treatment for a specific individual under particular circumstances (Paul, 1967). Moreover, it fits the aim of predicting “the optimal treatment for a given individual and the magnitude of the advantage” (van Bronswijk et al., 2021). This deeper understanding of precision not only points out the rough aims but proposes how these aims are meant to be achieved. Thus, among those who have articulated their opinions and work in the field of precision psychotherapy, our proposal is likely to find no strong opponents at first glance, suggesting that it is not obviously in conflict with what scientists in precision psychiatry actually do and want.

Despite the medical understanding and, more specifically, the psychiatric understanding of precision not being in direct conflict with our proposal for how to understand precision psychotherapy, one may worry that there is something about psychotherapy that resists being neatly subsumed under this framework—a discontinuity between psychotherapy and the rest of medicine that puts psychotherapy at odds with the above-sketched understanding. A particularly relevant instance of this worry shall be discussed here. This worry looks as follows. Psychotherapy in its current practice could either be considered the most precise medical form in existence or the most imprecise medical practice out there. The reason to believe it to be precise is that therapists, although having some general treatment-relevant models of how to psychologically understand psychiatric issues in mind, such as the SORKC Model (Kanfer and Saslow, 1965) in CBT or conflict theory in psychodynamic therapy (Schneider, Klauer, and Freyberger, 2008), usually engage with patients by tailoring conversation and exercises exclusively to the individual. On the other hand, the reason to consider psychotherapy extremely imprecise and, on a practical level, hard to become more precise is that most psychotherapists have a particular style of working or niche of practice and would be unable or at least unwilling to overcome their longstanding, in part perhaps habitual, approaches to working with patients and alter their fundamental approach to tailor to a specific patient. These two positions stand in tension with each other and each might evoke the intuition that our advocated working definition of precision psychotherapy is insufficient. Either because it misses the already existing precision in common therapeutic practice, or because it wants to develop psychotherapy into something, it will have a hard time realizing due to practical reasons. How should we handle this plausible worry related to the actual work of psychotherapists considering our proposal for a working definition of precision psychotherapy?^16^ Let us explain.

Psychotherapy in its earnest attempt to suit treatment to individual patients is in part guided by the attempt to be precise, and by doing so can rightfully claim for itself to work based on general treatment models that get specified to a certain degree for individual patients, or perhaps rather a specific set of psych-behavioral aspects of the patient that inform the specification of the general therapeutic model for the case of the patient. Such specification will plausibly lead to some precision, but in some parts it will not, so that there is still room for improvement. Considering this, the baseline precision from which psychotherapy is starting prima facie seems to be higher. However, it is not clear how much of this specificity indeed will stand the test of detailed scientific investigation. Such a study would focus on more conceptual clarity (increase of semantic precision), better understanding how certain valid assumptions are commonly made(causal precision) and whether the baseline assumptions of these models turn out to not only be of practical use but are also empirically plausible (epistemic precision). Hence, our working definition allows us to make some concessions to the current efforts of psychotherapists that both now and in the future that result in the empirical support to contribute to and maybe even embody a high level of precision as compared to other medical fields. Nevertheless, it also urges caution, to assume not automatically all specifications to be precision-focused and remain open for the evaluation of current therapeutic practice regarding its level of precision. This then also brings us to the other side of the apparent dilemma, the worry that psychotherapy may occur to be lost to imprecise practice due to practical concerns.

On the other side of the tension, we have the indicated personal practices of therapists. Such personal style aspects may concern many aspects. For example, whether therapists shake their patients’ hand or not and the way they end therapy sessions, whether they make jokes in therapy sessions, if they tend to be more confrontational or supportive, if they themselves tend to tear up if they feel strong empathy toward their patients, how much of their understanding of their patients’ problems they disclose to them, and many other topics, and whether they consider syndromal diagnostics of any relevance to the actual therapeutic work or not (Kind, 2023, 2025).

While, as expressed in the first presentation of the worry regarding personal practices, it might be beyond the therapist’s control to change some of their behaviors in treatment (e.g., showing certain mimical reactions or tearing up) or they might not want to change aspects of their practical work (e.g., shaking patients’ hands to greet them), this in itself does not necessarily make psychotherapeutic practice as it stands extremely imprecise, nor does it have to be an all-out hurdle for therapy to become more precise, if we consider the above argued working definition of precision. The first reason to not assume that personal style necessarily lowers or hinders precision is that many of them may be irrelevant or at least of very limited relevance to the precision of therapeutic practice. Whether the therapist shakes patients’ hand or the way a therapist lights their practice will prima facie not be a significant difference-maker between a success and failure of treatment as long as the therapeutic working relationship is otherwise intact, making it mostly irrelevant for our understanding of psychotherapy (not important to epistemic precision) and to the outcomes of therapy (not important for causal precision), while it also does not seem to be somehow relevant to a better conceptual grasp of therapy-related issues (not relevant to semantic precision). Hence, many aspects of personal style may be irrelevant to the precision of therapy so that therapists do not have to worry that precision therapy would be some technocratic requirement dictating every aspect of their work. However, plausibly there will be some personal styles that may be part of the therapy style of the therapist that these therapists may have habituated or want to hold on to that would indeed contribute to the imprecision of therapy. Let me take one example. While many therapists believe that transference interpretations are key drivers of the effectiveness of psychodynamic psychotherapy and some forms of dynamic therapy, such as Transference-Focused Psychotherapy (Clarkin and Kernberg, 2015), and even assume these interpretations to be the drivers of therapeutic success with some patient populations such as individuals with Borderline-Personality Disorder, some empirical investigations contradict these assumptions of the perhaps first disorder-specific psychodynamic therapy approach. Some research suggests that interpretations of transference phenomena in patients who show low levels of personality functioning, which is assumed by Clarkin and Kernberg to be typical for individuals with Borderline Personality Disorder, do not particularly benefit from or even show adverse outcomes, the more that transference interpretations are used (Høglend et al., 2006, 2008; Hersoug et al., 2014). If we assume this evidence to suggest the right conclusion, practicing Transference-Focused Psychotherapy with Borderline patients, opposed to what convicted therapists using this method believe, would not mean an increase in Causal Precision (i.e., leading to the use of interventions that lead to better clinical outcomes) and maybe even decrease the causal precision of their work by causing adverse effects. Moreover, this approach would lead to epistemic imprecision by assuming the wrong medical proposition that transference interpretations are of positive relevance to the treatment of borderline patients. Hence, if the conclusion supported by the work of Høglend and others were right, TFP therapists or those who made it one of their personal preferences to provide many transference interpretations with borderline patients would have to accept that their approach is not particularly precise, maybe even imprecise. This would then force them either to bite the bullet of knowingly working in a nonprecise and potentially even imprecise manner, or to give up some core commitments of their specific therapeutic approach.

Indeed, such changes may be hard to implement and face relevant resistance not only for rational reasons (e.g., evidence to the contrary) but also for emotional reactions due to cognitive dissonance one experiences when having done something in good faith for some time and then being told it was potentially harmful. However, such developments in medicine are not unique and are part of the process in all areas of medicine. From bloodletting to prescription practices of antipsychotic medications, there perhaps always were individuals and clinicians who resisted evidence or had to accept that what they did in practice or assumed about their area of expertise was wrong. As a result, the response to the worry that the personal style of therapists may render psychotherapy the most imprecise field, drawing on our account of precision, we can therefore answer that this worry is perhaps overstated, while not entirely without basis: Many aspects of personal practice will perhaps not be in tension with increasing the precision of one’s personal therapeutic preferences to any significant degree, however some may and perhaps will, so that some imprecision in psychotherapy due to personal or sub-group specific therapeutic preferences should be expected to be sources of imprecision and will have to be given up if one wanted to maximize precision.

To conclude, with our proposed working definition in mind, the following response to the worries that psychotherapy is either already a highly precise medical business or could be considered to be highly imprecise and hard to be made more precise can be given. It seems plausible that psychotherapy in many regards has already achieved a considerable level of precision by its focus on the individual psychological and social features of patients. However, it should at the same time be expected that the specification of therapy to adapt to the individual patients may not all turn out to be actual increases in precision when more research is done on the regarding aspects of practice and the relevant background assumptions. On the other hand, the worry that preferences and style in therapeutic practice are hopelessly imprecise and overall resistant to an increase in precision also seems to be too general. While it is to be expected that some aspects of personal practice are at odds with the aims of increasing precision, many aspects of personal preference in practice in psychotherapy will not be of concern or at least not highly relevant to the aim to increase precision in psychotherapy, thus not leading to a high amount of imprecision or in presenting its increase if continued. In the end, it seems that both worries have some validity, but that in light of our proposed understanding of precision they can be partly defused so that aiming for more precision in psychotherapy neither appears to be superfluous because it is already highly precise, nor seems to be practically impossible because it would require therapists to submit fully their work under a paradigm that would force them to give up all individuality they bring to their work.

## VI. AN APPLICATION CASE

Having presented our proposal in abstract terms in the last section, let us provide a more concrete idea of how increases in precision relevant to our understanding of precision psychotherapy may look in practice.^17^ To do this, we contrast two scenarios. In the first scenario, we imagine a patient receiving usual psychotherapeutic treatment in an outpatient setting. In the second scenario, the same patient receives treatment in the same setting, but augmented by aspects that increase the precision of the therapeutic treatment as intended by our working definition. In this scenario, we present examples of increases in precision along each trajectory of precision we mentioned: semantic, epistemic, and causal precision. The precision-increasing elements we introduce are not purely fictional; these are approaches currently being developed in research and, if progress continues, become features of routine therapeutic work in the not-so-distant future.

Let us begin with the first scenario. Imagine that a patient comes to an outpatient treatment practice, initially reporting symptoms typical of depression. The psychotherapist, following current good diagnostic practices, conducts a diagnostic interview with the patient and, if the therapist is not a medical doctor, refers the patient to a medical doctor to ensure no non-psychiatric medical condition, potentially causing the symptoms to be overlooked. Concluding the diagnostic procedures, the psychiatrist determines that the patient’s symptoms support the syndromal diagnosis of major depression according to ICD-11. Assuming roughly equal average efficacy for both major psychotherapeutic approaches—cognitive-behavioral therapy (CBT) and psychodynamic therapy (see, e.g., Smith and Hewitt, 2024)—the psychotherapist explains the options to the patient and, based on the patient’s preferences, decides to start psychodynamic psychotherapy.

During therapy, the therapist follows the standard strategies and techniques of this approach, addressing the patient’s issues, which in psychodynamic therapy are often conceptualized in terms of internal motivational conflicts. The goal is to understand and potentially resolve these conflicts at a conscious level, develop psychological capacities to better handle emotions and thoughts, and deal with external adverse life events, all intended to allow symptom reduction (see, e.g., Gabbard, 2014). Roughly halfway through the planned sessions, the psychotherapist and the patients review the therapy’s progress. They are pleased to see some improvement in the patients’ symptoms and attitudes. They continue with the treatment until the end, concluding with the patients in partial remission and considerable improvement in their quality of life.

Many outpatient treatments follow a similar pattern to that sketched in this scenario. As we see in this example, there is not a full absence of precision; therapy is not a random process. This is no surprise since, in our understanding, precision is not an all-or-nothing phenomenon but the flip side of reducing different forms of indeterminacy, which usually occurs gradually. Let us consider the presence of precision in the first scenario along the three trajectories of precision we proposed. Precision in the sense of semantic precision is present through the classification of the patient’s condition as a certain disorder. Causal precision is present by specifying a treatment under the assumption that the outcome using this treatment is more likely a remission than using an empirically less supported or no treatment. Finally, there is also epistemic precision related to treatment choice, namely, the acceptance of medical facts assumed in the process, that is, that CBT and psychodynamic therapy can be assumed to lead to roughly equal outcomes in treating depression on average.

However, though the first scenario shows that psychotherapy as currently practiced is not an entirely imprecise procedure, there is room for improvement on all trajectories of precision, using a broad scope of biological, social, and psychological information. Hence, there is room to make therapy more precise. Let us look at a second scenario to consider what these increases in precision along the trajectories of semantic precision, epistemic precision, and causal precision may look like. For this, we discuss the subtyping of depression diagnosis, predicting the likelihood of better response to CBT or psychodynamic therapy, likelihood of non-response to therapy, and leveraging knowledge on the use or avoidance of specific psychotherapeutic interventions.

Imagine the very same patient coming to outpatient treatment, reporting depressive symptoms. However, already at the outset of the diagnostic procedure, things could differ. The depression fulfills the criteria of major depression. Rather than just giving this diagnosis and providing one of the broadly equally effective psychotherapies we have at our disposal, psychotherapists using some of the ways to increase precision currently being developed in research might proceed differently. In addition to providing an ICD diagnosis, they might further subclassify the depressive disorder into one of the subtypes under investigation, which could form the basis for more specific targeted treatments in the future, possibly based on MRI or other brain activity data (see, e.g., Yang et al., 2021; Jensen et al., 2023). Engaging in such subclassification would mean an increase in semantic precision compared to scenario one by refining psychiatric classifications.

Furthermore, the clinician might use the currently developed Personal Advantage Indexes as part of the initial assessment. These indexes gather data about psychological, social, and medical history factors and predict which of the general therapies (e.g., CBT or psychodynamic therapy) would be most beneficial for the specific patient (see, e.g., Røssberg et al., 2021; Bruijniks et al., 2022). The clinician may also gather information to determine how likely the patient is to be a non-responder to psychotherapy (i.e., a patient who does not show meaningful improvements) based on predictions from gathered data. In the future, this may be possible using psychological, social, or biological data (see, e.g., Cuijpers et al., 2021; Chekroud et al., 2021). This information would be relevant for discussing the prospects of success with the patient or considering additional psychopharmacological treatment. Gaining such patient-specific insight would mean an increase in causal precision compared to the generic procedure in scenario one, because it provides better insight into how likely it would be for treatment to help the patient and which treatment is most likely to help.

Finally, an aspect that might become more precise is the identification of specific interventions within the treatment. For example, it might become more common to determine which interventions are more beneficial for which patients. One example emerging from research on intervention in psychodynamic psychotherapy concerns the intervention of interpretation. While it has long been common sense that transference interpretations are key drivers of the effectiveness of psychodynamic psychotherapy, recent research suggests that interpretations of transference phenomena in patients who show low levels of personality functioning (those who are “better ego-structured,” as psychodynamic therapists may say) do not benefit from or even show adverse outcomes from these kinds of interpretations. However, these interpretations tend to be beneficial for individuals with higher personality functioning (see Høglend et al., 2006; Høglend et al., 2008). Applying this insight to our imagined depressed patient could mean that in addition to the depression diagnosis, psychological and social information that helps determine the level of the patient’s personality functioning would become relevant to decide whether transference interpretations would be useful for the treatment. By correcting commonly held views, like the assumption that transference interpretations are generally useful, research can increase epistemic precision by correcting assumed medical facts. This, in turn, reduces causal indeterminacy, because the increase in epistemic precision corrects an assumption about the benefits of treatment options.

To sum up, considering the proposed precision-increasing elements for the second scenario, the therapy process may look different in some regards. The initial diagnostic efforts are enriched by a more precise subtyping of the depression diagnosis based on bio-psych-social information. Treatment choices (CBT or psychodynamic therapy) are informed by a Personal Advantage Index, and insight into personal likelihood of non-response is considered and can be discussed with the patient. Moreover, if a psychodynamic therapy is chosen, specific recommendations on interventions to use or avoid can be leveraged, based on collected psycho-social information about the patient. If these changes are implemented, the rest of the conduct of the treatment, its evaluation halfway through therapy, and its further conduct until the end, hopefully leading to remission, can still look the same unless further precision-increasing elements concerning these treatment steps are introduced. I focus for now on the above-presented elements only to provide an example of the increase in precision between scenario one and scenario two along each trajectory: This was done in terms of semantic precision by introducing the imagined option of diagnostic subtyping, causal precision by the imagined option of prediction methods to determine the optimal treatment method and non-response likelihood, and epistemic precision (also affecting causal precision) by adopting the medical assumption that certain interventions, rather than being beneficial to all depression patients, are helpful to some, but non-beneficial or even harmful to others. Now that we have a more practical idea of how our working definition of precision psychotherapy would map onto actual changes in therapeutic practice, we come to our conclusions.

## VII. CONCLUSION

In this paper, we take significant strides towards a more rigorous understanding of precision psychotherapy. We accomplish this by conducting a thorough review of the ongoing discourse on precision medicine (Section II) and by introducing novel insights on how to conceptualize precision in medicine (Sections III and IV). Building on this foundation, we propose a new and robust definition of precision psychotherapy (Section V) and discussed an application of the proposed understanding (Section VI). This definition not only aligns with what we argue constitutes precision medicine in general but is also in line with the limited existing discussions within the emerging field of precision psychotherapy. We believe this definition will serve as a solid basis for further conceptual debates and offers practical utility, aiding entities such as funding agencies and lawmakers in determining what qualifies as research in precision psychotherapy. Furthermore, it empowers researchers to discern what research can be classified as precision psychotherapy and what cannot.

## Footnotes

1 “People frequently cite the 1990s as the era for redefining mental disorders as brain disorders. While this conceptual shift was important, we now realize the greater importance of developing new tools: imaging techniques for quantitative studies of brain structure, function, and chemistry, as well as other comprehensive tools for mapping DNA and RNA. (…) These advances ushered in a decade of discovery that brings us to 2010” (Dana Foundation, 2010).

2 To just give one example of the early achievements of the pharmacogenetic instance of personalized medicine, let us consider an improvement in the treatment of HIV by the drug Abacavir. Abacavir was a relatively safe drug for most patients; in some patients, however (6%), it caused a life-threatening allergic reaction. This allergic reaction, however, could be correlated with a single genetic variant of the major histocompatibility complex class I (HLA-B*5701) (Hetherington et al., 2002; Mallal, 2002). Subsequently, this discovery allowed them to implement screening procedures for HIV patients for the relevant genetic variant before the administration of the drug.

3 The reader may wonder about the appearance of “personalized” instead of “precision” in this journal’s names. In fact, both terms are often used synonymously (see, e.g., Fröhlich, 2018), as is the case with these journals.

4 The interested reader may find more details on the history of precision medicine, its (dis)continuity with previous developments in medicine in Ruaño (2004), Steele (2009), Longo (2013), Michl (2015), Perlman et al. (2016), and Lee et al. (2019). For debates on how much progress was actually made through precision medicine and how much more should be expected, see, e.g., Hedgecoe (2004), Green and Guyer (2011), Chabner et al. (2013), Maughan (2017), Fröhlich et al. (2018), and Denny and Collins (2021).

5 By making more extensive use of psychosocial and environmental information, this approach follows in the footsteps of the Biopsychosocial model proposed by Engel (1977). While the early days of the Biopsychosocial model were in principle often regarded as highly plausible, it was plagued by problems of poor scientific understanding of each of the domains and how to integrate them. This has led some authors to recently claim that the Biopsychosocial model has extensive limitations or has even failed (see, e.g., Benning, 2015; Roberts, 2023). Others, however, see advances in precision medicine, and especially psychiatry, now making sound use of biopsychosocial information as the most recent attempt to capitalize on the full potential a non-reductive Biopsychosocial approach to illness may have (e.g., Bolton and Gillette, 2019).

6 At this point, the reader may wonder why we only talk about types of information used in precision medicine when discussing its means and not its methods as well. We exclude methods from our discussion simply because the sheer range of potential and actual methods employed to achieve the aims of precision medicine across various medical disciplines is so vast that attempting to address them all would be impractical. Additionally, it does not appear that any specific method is excluded from use in achieving precision, nor does there seem to be ongoing debate about this matter. Undoubtedly, in contemporary times, machine learning methods applied to large datasets of patient information are assuming an increasingly prominent role in medical research, and there is optimism that this may lead to a significant breakthrough (e.g., Love-Koh et al., 2018; Johnson et al., 2021). However, no particular method appears to be definitive or central to current discussions on what defines precision medicine.

7 We continue to use the convention of using “personalized” and “precision” medicine interchangeably. However, it is important to note that this practice is not universally accepted. The National Research Council (NRC), for instance, prefers not to use them interchangeably. While both terms are sometimes used synonymously, the term "personalized medicine" has been used to convey the idea that unique treatments can be designed for each individual. Due to this, the NRC believes that the term "precision medicine" is a better choice to describe the large-scale medical movement they have in mind (NRC, 2011, 125). Similar concerns about the term "personalized" arise repeatedly in the literature. For instance, Feiler et al. point out that the focus on the personal in personalized medicine may generate a mystique of unrealizable promises and expectations about medicines and treatments perfectly tailored to each individual person (Feiler et al., 2017, 4).

8 Of course, someone who would cling to any a priori reason why precision should only be restricted to the narrow notion might think otherwise and not be convinced by this presentation. In other words, one would have to apply what Tabb, Lemoine pointed out, namely that “Given the amorphous role of the concept of precision, either precision medicine must be understood to mean different things in different fields or, if its usage in oncology is taken to be paradigmatic, psychiatry (and likely other fields as well) must be admitted to be imprecise” (2022, 194). Since the notion of precision in itself as spelled out earlier does not commit one to the narrow or broad notion of precision per se, we for our part go with the first option.

9 By proposing an alternative understanding of precision that is not the one assumed in the case of measurement, we do not mean to suggest that this kind of precision is irrelevant to medicine or psychotherapy in general. If we, for example, conduct investigations of medical or psychotherapeutic interventions where we measure some aspects of these, having precise measures is, of course, crucial. However, we contend there is a difference between the precision needed in scientific investigation, including medical interventions, and the way an increase in precision of these interventions themselves should be understood.

10 For a more detailed discussion on metaphysical indeterminacy, see Williams (2008).

11 For a longer discussion of different kinds of semantic indeterminacy, see Sorensen (2022).

12 At this point, one may wonder whether the reduction of semantic indeterminacy, and thus the increase of precision in how medicine refers to its objects, may occur in a relevant manner in the context of medicine. For now, we proceed by discussing the different kinds of indeterminacy we consider relevant and ask for patience. In Section 4, we discuss examples for each kind of indeterminacy reduction, including the reduction of semantic indeterminacy in medicine.

13 This aspect of precision, for example, occurs in the National Institute of Health’s (NIH) discussion of what precision medicine is (NIH, 2022), in that the NIH paraphrases the position of the American Research Council, saying that precision medicine among others is about “the ability to classify individuals into subpopulations that differ in their susceptibility to a particular disease.”

14 This example is taken from the research of Kresovich et al. (2020).

15 This aspect of increase of precision is, e.g., pointed out by Cherny et al. (2014, 1): “Rather than having medications recommended on the basis of diagnosis and staging, ‘personalized medicine’ suggests that tailored treatments based on assessment of biological parameters of the individual or the underlying disease can improve patient outcomes by identifying those patients most likely to benefit from specific therapies and, simultaneously, diminishing the use of medications for patients who can be predicted not to derive benefit from them.” Also, this aspect appears in the earlier referenced statement of the NIH saying that precision medicine is among others interested in “prognosis of those diseases they [patients] may develop, or in their response to a specific treatment” so that “preventive or therapeutic interventions can then be concentrated on those who will benefit, sparing expense and side effects for those who will not” (NIH, 2022).

16 We are grateful to the reviewers for bringing up this point.

17 We are grateful to the reviewers for bringing up the importance of an illustrative example.
